# Research on Multiscale Modeling and Experiment of CFRP Milling

**DOI:** 10.3390/ma16206748

**Published:** 2023-10-18

**Authors:** Jing Ni, Haishan Liu, Zhi Hong, Aihua Meng, Mingfan Li

**Affiliations:** 1 School of Mechanical Engineering, Hangzhou Dianzi University, Hangzhou 310018, China; nj2000@hdu.edu.cn (J.N.); lhs212010066@hdu.edu.cn (H.L.); mengah@hdu.edu.cn (A.M.); 2 School of Mechanical and Automotive Engineering, Zhejiang University of Water Resources and Electric Power, Hangzhou 310018, China

**Keywords:** CFRP milling, multiscale FE model, thermal-mechanical coupling, cutting parameters, material removal mechanism

## Abstract

High-quality milling of carbon fiber reinforced polymer (CFRP) composites is of great importance for the high-performance manufacturing of structures made of this hard-to-machine material. In this paper, a multiscale finite element (FE) model, considering the thermal–mechanical coupling effect, was developed to simulate the milling process and reveal its material removal mechanism. The corresponding milling experiments were conducted to validate the simulated cutting forces and temperature, which were in good agreement with the experiment results. In the macroscale model, the Hashin failure criteria were used to estimate the failure of the composites. In the microscale model, the fibers, matrix, and the fiber–matrix interface were modeled separately, to investigate the mechanisms of material removal behavior during milling, among fiber breakage, matrix cracking, and fiber–matrix debonding. Based on the macroscale numerical and experimental results, the higher cutting speed was demonstrated to improve the surface quality of CFRP milling. According to the results from the microscale model, the material removal mechanism varies depending on the orientation of the fibers and can be divided into four stages. The outcome of this work provides guidelines to further investigate optimal manufacturing parameters for the milling of CFRP composites and their cutting mechanisms.

## 1. Introduction

Carbon fiber reinforced polymer (CFRP) composites have found extensive use in the aerospace industry due to their excellent strength-to-weight ratio, resistance to fatigue, and low thermal expansion [1]. At present, some key structural components in aircraft are made of CFRP, such as the wings, fuselage, and stabilizer sections. High-performance machining operations, such as trimming and milling, are unavoidable when manufacturing components with intricate shapes [2]. However, CFRP is known for its challenging machinability. The machining of CFRP with toughened resins or high-strength reinforced fibers might easily lead to defects, including fiber pull-out, delamination, matrix–fiber debonding, or sub-surface damage, which hinders the attainment of the desired quality and strength requirements [3]. Furthermore, considering that the cutting temperature is likely to surpass the resin’s glass transition temperature, leading to the degradation of the matrix, it is essential to acknowledge the thermal impact throughout the cutting procedure [4]. Therefore, it is crucial to investigate the material removal mechanisms of milling CFRP within the thermal effect and assess the impact of the processing parameters on the inflicted damage.

There are some experimental and analytical modeling studies for CFRP milling and material removal mechanisms. Ozkan [5] discussed material failures and tool wear mechanisms in CFRP milling, and methods to avoid machined damage were discussed. In Zhang’s study [6], high-speed side milling experiments were conducted to assess the influence of up/down milling and machining parameters on cutting forces and the surface quality of CFRP. The findings indicated that up-milling was preferable for removing CFRP material. Knápek [7] found tool geometry had a significant influence on the tool’s wear, cutting forces, and machined edge roughness when milling CFRP. Song [8] established a surface roughness prediction model, and carbon fiber distribution was considered. The formation mechanisms of surface roughness were first elucidated in milling CFRP with 90° fiber orientation. Zan [9] investigated the effects of temperature on the removal mechanism and surface integrity of machining metal matrix composites (MMCs): the machinability under heat/cryogenic assistance was discussed separately.

Compared to experimental studies and analytical models, finite element simulation is a simple method to visualize material removal and damage formation processes with detailed output variables at desired geometric scales and locations. Wang [10] and Ali Kouka [11] used the 3D Hashin progressive damage model to model the failure of CFRP. In a study conducted by Su [12], they analyzed the influence of cutting velocity on the cutting process and the formation of damage in the orthogonal cutting of CFRP using simulation. The findings indicated a significant decrease in fiber deformation in the impacted area before fiber fracture with an increase in cutting speed. Liu [13] created a model for plastic composites made of CFRP that considered the random arrangement of carbon fibers. The model was employed to study the micro-cutting mechanism and assess the quality of the surface. Seyedbehzad Ghafarizadeh [14] conducted a study on the flat-end milling process of unidirectional CFRP. The study focused on analyzing the cutting forces, the process of chip formation, and the resulting damage during machining. Wang [15] established a three-dimensional microscopic milling model for CFRP that predicted interlaminar fractures and cracks.

However, the FE models do not consider the effect of temperature on the material removal behavior, which limits our understanding the real machining process. The cutting heat not only causes thermal damage but also worsens mechanical damage in the cutting area [16]. To ensure an accurate analysis of the temperature dispersion and the possible impact of heat on the failure of CFRP, it is crucial to incorporate the thermal–mechanical coupling effect in the simulation of CFRP machining. Cheng [17] utilized a straightforward and effective way to describe the thermal–mechanical interaction, developing a microscale thermal–mechanical coupling model based on plastic energy dissipation and the generation of heat through friction during the cutting process. In Xu’s study [18], a microscale model considering thermal–mechanical coupling was developed to investigate machining the CFRP with ultrasonic vibration. According to the results, the cutting force was minimally affected by the cutting temperature, but the temperature influenced the material removal process significantly. Sheikh-Ahmad [19] conducted studies to calculate the heat partition in CFRP edge trimming and orthogonal cutting, respectively. Sheikh-Ahmad simulated transient heat transfer issues in the lamina and cutter independently using an iterative inverse heat transfer approach and calculated the volume of heat flux injected into each. In Yan’s study [20], a combination of experiments and simulations was used to examine the dissipated energies related to various failure modes and friction. It was found that these energies varied with the fiber orientations. Sensitivity analysis was also conducted to provide further insights.

Although the above FE models were commonly used to study fiber-reinforced composite cutting processes, they had limitations due to their micromechanical approach. Microscale modeling involves complex calculations and requires exact details about fibers, matrices, and interfaces, which can result in long computation times. As a result, some researchers have turned to macro mechanical approaches to model this process. In a study by Han [21], CFRP was regarded as an equivalent homogeneous material with temperature-dependent properties. The objective of the research was to elucidate the mechanisms of removing material from UD–CFRP composites during orthogonal cutting, by employing thermal and cryogenic pretreatments. Lasri [22] and Zenia [23,24] built the macroscopic simulation model, respectively, to analyze the cutting of CFRP. Lasri found that the Hashin criterion provided values that were closer to the experimental results, while Zenia’s model considered the plasticity of the workpiece and showed that the minimum cutting force was obtained when the fiber orientation was near 15°, and that the maximum value was observed near 75°. Wang [25] found that nearly all of the thermal energies in orthogonal cutting unidirectional CFRP were transmitted to the workpiece and chips. The 90° and 135° fiber orientation CFRP were observed as the highest partition.

However, the models mentioned above are developed from either a macroscopic or microscopic single viewpoint, resulting in limited comprehension. Multiscale models can simulate the damage that initiates and accumulates from the quantum to the microscale, ultimately leading to failure at the macroscale [26]. Therefore, it is crucial to comprehend the removal behaviors of materials at the macroscale and microscale to accurately quantify damage after machining and explain cutting mechanisms. Wang [27] developed both macroscopic and microscopic models to analyze the material removal process at various levels, which included material constitutive, failure initiation, and damage evolution law. In the study conducted by Hassouna [28], the macro mechanical model examined the impact of CFRP mechanical characteristics, fracture energy, and hourglass control. Additionally, a micromechanical model was used to study the chip formation mechanisms by treating the composite constituents separately and comparing them with the former model. Mkaddem [29] developed a macro–micro model that provided improved accuracy in predicting global responses, while also allowing for the analysis of micromechanical mechanisms, such as local damage. The results demonstrated that this model yielded better predictions of cutting and thrust forces under different fiber orientations.

In summary, despite considerable works having been performed to investigate CFRP cutting, only a limited number of them have taken into account the thermal–mechanical coupling effect. Additionally, these studies have not adequately investigated the cutting mechanism of CFRP through multiscale analysis. Furthermore, it should be highlighted that the selected cutting parameters in most models do not accurately represent real-world processing conditions. To fill this gap, this paper aims to present a thermal–mechanical model at multiple scales, to simulate the cutting process and reveal the CFRP removal mechanisms. Corresponding experiments will be conducted to examine the model prediction’s reliability and accuracy. The structure of the paper is as follows: Section 2 illustrates the FE modeling, including the framework of the multiscale model on the milling CFRP composites, materials’ constitutive definitions, and corresponding failure criteria. Section 3 presents the setup of the experiment. Section 4 discusses the influence of the machining parameters on the cutting force and temperature and explains the material removal mechanisms based on microscale model results. The last section is the conclusion.

## 2. Multiscale Modeling

### 2.1. Framework of the Multiscale Model

**Figure 1:**
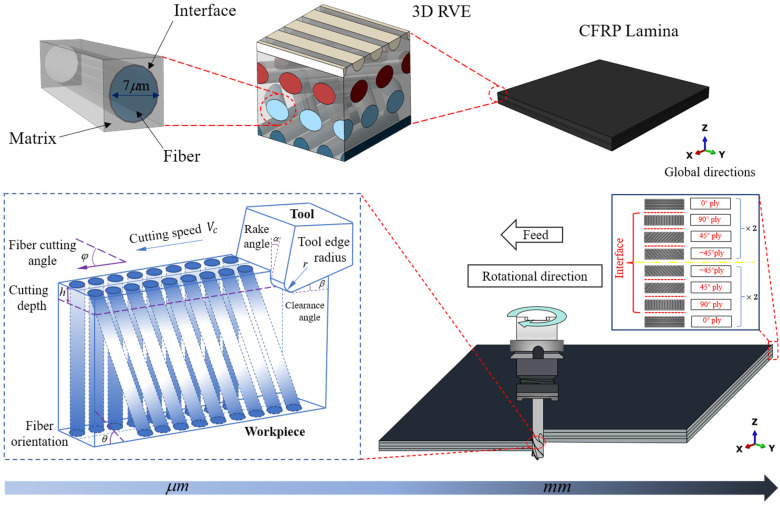
The framework of the multiscale model.

Since composites naturally exhibit multiscale features, the multiscale FE approach is an excellent tool to describe the properties of composites and illustrate the material removal processes. For CFRP composites, the CFRP lamina is composed of thousands of representative volume elements (RVE) in mesoscale. The RVE consists of an organized dispersion of fibers embedded in the matrix and an interface between them, which is sufficient in size to maintain the characteristics of various materials. In addition, the cohesive zone modeling (CZM) method is frequently applied to simulate the behavior of traction separation between fiber, matrix, and ply-to-ply interfaces. The multiscale framework of cutting CFRP composites is depicted in Figure 1. Firstly, the macroscale thermal–mechanical coupling finite element model, based on the EHM method, is built to simulate the milling process of CFRP lamina, and the simulation cutting forces and temperatures are validated by experiments. Secondly, a 3D RVE thermal–mechanical coupling model is established, which simulates CFRP composite removal mechanisms under the temperature obtained from the results of experiments.

### 2.2. Heat Generation and Transfer during Milling CFRP

In metal cutting, there are three potential heat sources:

(1) the material deforms and breaks;
(2) there is friction between the rake face of the tool and the chips;
(3) there is friction between the flank face of the tool and the machined surface of the workpiece.

**Figure 2:**
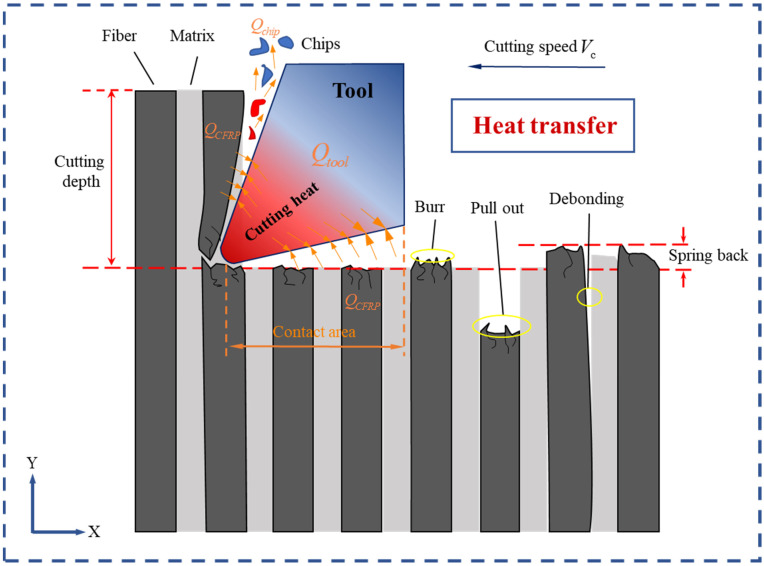
Heat generates and transfers during the cutting of CFRP.

However, because of the brittle characteristic of CFRP composites, sharp crushing and fracturing of carbon fibers occur with little matrix material deformation. The development of powder chips indicates the main removal mode is fracture propagation. As a result, the amount of heat generated from plastic deformation is almost negligible when cutting CFRP composites. Alternatively, the primary source of heat generation can be attributed to the second and third points mentioned above [30]. According to Refs [31,32], almost all of the mechanical energy can be transformed entirely into thermal energy. Figure 2 illustrates the division of heat energy into three components: the tool, the chips, and the CFRP workpiece, where, $Q_{total}$ is the total heat energy around the cutting contact zone, and the heat energy diffusing into the tool of the CFRP workpiece, and the chips, separately.

CFRP composites show anisotropic thermal properties because of their particular composition. To achieve chip formation during the cutting of CFRP composites, it is essential to obtain external energy to overcome the material strength. This process generates a considerable amount of heat due to the friction between the tool and chips, which leads to a change in CFRP thermal and mechanical properties. To accurately model this process, it is essential to simulate the generation and the transfer of heat. The fundamental cutting temperature evolution algorithm is shown in Equation (1) [33].

(1) $$\frac{\partial }{\partial x}(k_{1}\frac{\partial T}{\partial x})+\frac{\partial }{\partial y}(k_{2}\frac{\partial T}{\partial y})+\frac{\partial }{\partial z}(k_{3}\frac{\partial T}{\partial z})+Q(t)-\rho C\frac{\partial T}{\partial t}=0$$

where $k_{1}$, $k_{2}$, and $k_{3}$ respectively represent the thermal conductivity in the X, Y, and Z directions shown in Figure 1. In this paper, $k_{2}=k_{3}$, ρ, and C are the density and the specific heat, respectively. The total heat generation is Q(t). In addition, the temperature gap between the tool and the workpiece causes heat to transfer through conduction and diffusion between the elements, which can be measured and analyzed by Equation (2) [34,35]:

(2) $$\left\{\begin{array}{l} q_{w}=h_{c}(T_{t}-T_{w})-\alpha \mu pv^{\prime }\\ q_{t}=-h_{c}(T_{t}-T_{w})-(1-\alpha )\mu pv^{\prime } \end{array}\right.$$

where $q_{w}$ and $q_{t}$ represent the heat flux density of the workpiece and tool, respectively, p is the pressure on the contact area, μ is the friction coefficient, $v^{\prime }$ is the relative speed, α is the heat partition coefficient, $h_{c}$ is the thermal contact conductance coefficient, and $T_{t}$ and $T_{w}$ are the temperature of the tool and the workpiece, respectively.

### 2.3. The Macroscale FE Model

#### 2.3.1. Macroscale Material Constitute Models

The ABAQUS v2022 software was utilized to develop the macroscale FE model, employing the equivalent homogeneous method (EHM). The CFRP composite was regarded as an anisotropic material with properties varying along the fiber orientation (direction 1) and perpendicular to it (directions 2 and 3). The material was presumed to maintain a linear elastic behavior until failure. The 3D Hashin criteria was used to model the progressive damages of the CFRP by a user subroutine VUMAT. There are four failure modes included in Hashin criteria, as follows [36]:

Fiber tension $\left(\sigma_{11}>0\right)$,

(3) $$\xi_{Ft}={\left(\frac{\sigma_{11}}{X_{T}}\right)}^{2}+\frac{\sigma_{12}^{2}}{S_{12}^{2}}+\frac{\sigma_{31}^{2}}{S_{13}^{2}}-1\geq 0$$

Fiber compression $\left(\sigma_{11}\leq 0\right)$,

(4) $$\xi_{Fc}={\left(\frac{\sigma_{11}}{X_{C}}\right)}^{2}-1\geq 0$$

Matrix cracking $\left(\sigma_{22}+\sigma_{33}>0\right)$,

(5) $$\xi_{Mt}=\frac{1}{Y_{T}^{2}}{\left(\sigma_{22}+\sigma_{33}\right)}^{2}+\frac{1}{S_{23}^{2}}\left(\sigma_{23}^{2}-\sigma_{22}\sigma_{33}\right)+\frac{1}{S_{12}^{2}}\left(\sigma_{12}^{2}+\sigma_{31}^{2}\right)-1\geq 0$$

Matrix crushing $\left(\sigma_{22}+\sigma_{33}\leq 0\right)$,

(6) $$\begin{array}{ll} \xi_{Mc} & =\frac{1}{Y_{c}}\left[{\left(\frac{Y_{C}}{2S_{23}}\right)}^{2}-1\right]\left(\sigma_{22}+\sigma_{33}\right)+\frac{1}{4S_{23}^{2}}{\left(\sigma_{22}+\sigma_{33}\right)}^{2}+\frac{1}{S_{23}^{2}}\left(\sigma_{23}^{2}-\sigma_{22}\sigma_{33}\right)\\ & +\frac{1}{S_{12}^{2}}\left(\sigma_{12}^{2}+\sigma_{31}^{2}\right)-1\geq 0 \end{array}$$

where $X_{T}$ and $X_{C}$ donate tensile strength and compressive strength in direction 1; and $Y_{T}$ and $Y_{C}$ donate tensile strength and compression strength in direction 2, respectively. $S_{12}$, $S_{23}$, and $S_{13}$ represent shear strength.

In this model, σ is the component of effective stress tensor, to assess the damage initiation, based on the following:

(7) $$\sigma =M\hat{\sigma }$$

where $\hat{\sigma }$ is true stress and M is damage factor, as follows:

(8) $$M=\left[\begin{array}{ccc} \frac{1}{\left(1-d_{f}\right)} & 0 & 0\\ 0 & \frac{1}{\left(1-d_{m}\right)} & 0\\ 0 & 0 & \frac{1}{\left(1-d_{s}\right)} \end{array}\right]$$

where $d_{f}$, $d_{m}$, and $d_{s}$ are internal damage variables characterizing the fiber, matrix, and shear damages, respectively. These variables are obtained from Equation (9) to Equation (11).

(9) $$d_{f}=\left\{\begin{array}{c} \begin{array}{ccc} d_{f}^{t} & if & \sigma_{11}>0 \end{array}\\ \begin{array}{ccc} d_{f}^{c} & if & \sigma_{11}\leq 0 \end{array} \end{array}\right.$$

(10) $$d_{m}=\left\{\begin{array}{c} \begin{array}{ccc} d_{m}^{t} & if & \sigma_{22}+\sigma_{33}>0 \end{array}\\ \begin{array}{ccc} d_{m}^{c} & if & \sigma_{22}+\sigma_{33}\leq 0 \end{array} \end{array}\right.$$

(11) $$d_{s}=1-\left(1-d_{f}^{t}\right)\left(1-d_{f}^{c}\right)\left(1-d_{m}^{t}\right)\left(1-d_{m}^{c}\right)$$

where $d_{f}^{t}$, $d_{f}^{c}$, $d_{m}^{t}$, and $d_{m}^{c}$ are damage variables which represent fiber tension, fiber compression, matrix tension, and matrix compression, respectively.

#### 2.3.2. Macroscale Model Setup

**Figure 3:**
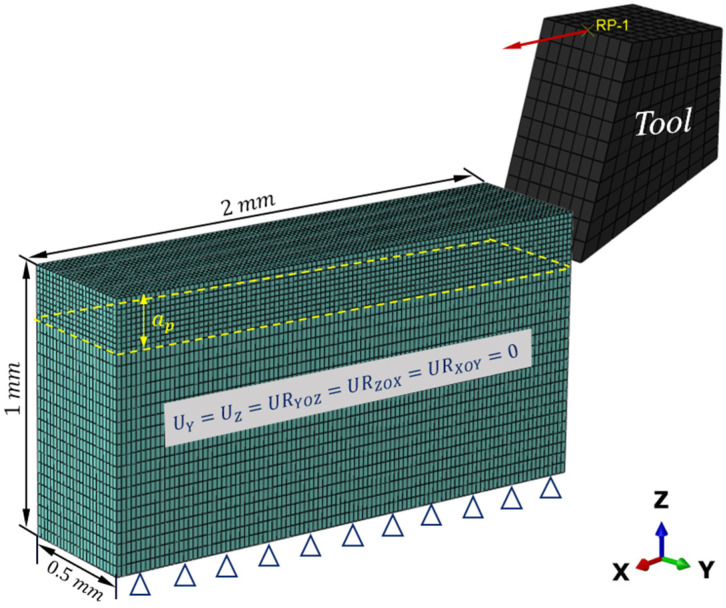
Macroscale geometric model.

The geometric model in macroscale is shown in Figure 3. To improve computational efficiency, the workpiece model size is simplified. The CFRP workpiece has a length of 2 mm, a height of 1 mm, a single layer thickness of 0.125 mm, and a total of four layers. The bottom and two sides of the workpiece are fixed. The cutting depth is 0.2 mm. To prevent the excessive distortion of elements, the meshes of the cutting area of the workpiece are refined to 0.02 mm and the other areas are meshed by 0.04 mm. Thermally coupled brick elements (C3D8RT) are used to mesh both the workpiece and the tool. The tool is treated to become a rigid body. The displacement and rotation of the tool are controlled by the reference point RP. The tool is defined to move at the cutting speeds set out in the experiments. The model assumes that the initial temperature of both the workpiece and tool are 23 °C and neglects the heat loss to the environment. The model of coulombic friction is employed to explain the interaction between the cutting tool and the workpiece, with a consistent friction coefficient of 0.3. In addition, a self-interaction requirement is implemented to avoid the occurrence of overlap between eroded components of the workpiece. The material parameters utilized in the macroscale model are identical in the experiment, provided by the manufacturer, as shown in Table 1.

### 2.4. The Microscale FE Model

#### 2.4.1. Microscale Material Constitute Models

##### Carbon Fiber

In this model, carbon fiber is seen as a transversely isotropic elastic material. Due to its brittle nature and lack of plastic deformation, the maximum stress criterion is used to determine its failure, which is also defined by a user subroutine VUMAT. This occurs when the element stress surpasses the corresponding strength in any given direction. Damage is described as follows:

Tensile loading in a longitudinal direction ($\sigma_{11}\geq 0$)

(12) $$d_{t1}^{f}=\frac{\sigma_{11}}{X_{t}}$$

Compressive loading in a longitudinal direction ($\sigma_{11}<0$)

(13) $$d_{c1}^{f}=\left|\frac{\sigma_{11}}{X_{c}}\right|$$

Tensile loading in a transverse direction ($\sigma_{22}\geq 0$ or $\sigma_{33}\geq 0$)

(14) $$d_{t2}^{f}=\frac{\sigma_{22}}{Y_{t}},d_{t3}^{f}=\frac{\sigma_{33}}{Y_{t}}$$

Compressive loading in a transverse direction ($\sigma_{22}<0$ or $\sigma_{33}<0$)

(15) $$d_{c2}^{f}=\left|\frac{\sigma_{22}}{Y_{c}}\right|,d_{c3}^{f}=\left|\frac{\sigma_{33}}{Y_{c}}\right|$$

Shear loading (in-plane) $(\tau_{12}>0$ or <0)

(16) $$d_{s12\pm }^{f}=\left|\frac{\tau_{12}}{S}\right|$$

Shear loading (out-plane)

(17) $$d_{s23}^{f}=\max\left\{\left|\frac{\tau_{13}}{S}\right|,\left|\frac{\tau_{23}}{S}\right|\right\}$$

##### Epoxy Matrix

In this model, the epoxy matrix is assumed to exhibit isotropic and elastoplastic behavior. Based on extensive engineering experience, the Johnson–Cook constitutive structure is capable of effectively describing the dynamic mechanical behavior of a wide range of metal materials as well as certain resin materials [12]. As a result, Johnson–Cook is utilized to assess the dynamic constitutive structure of epoxy resin during the cutting process.

(18) $$\sigma =\left(A+B\varepsilon^{n}\right)\left(1+C\ln\frac{\dot{\varepsilon }}{{\dot{\varepsilon }}_{0}}\right)\left[1-{\left(\frac{T-T_{r}}{T_{m}-T_{r}}\right)}^{m}\right]$$

where A, B, n, C, and m, are the coefficients determined by experiments. $T_{r}$ and $T_{m}$ are the room temperature and the melting temperature. σ and ε are equivalent stress and equivalent plastic strain. $\dot{\varepsilon }$ and ${\dot{\varepsilon }}_{0}$ are corresponding strain rates.

The shear failure criterion is used to predict its failure. The shear failure criterion postulates that the equivalent plastic strain at the initiation of damage is dependent on the shear stress ratio and the strain rate.

(19) $${\bar{\varepsilon }}_{S}^{p}(\theta_{s},{\dot{\bar{\varepsilon }}}^{p})$$

where $\theta_{s}=\frac{\left(q+k_{s}p\right)}{\tau_{max}}$ is the shear stress ratio, $k_{s}$ are material properties, $\tau_{max}$ is the maximum shear stress, q is the von Mises stress, and p is compressive stress. When the following formula is satisfied, the element fails and is deleted.

(20) $$\omega_{s}=\int \frac{d{\bar{\varepsilon }}^{p}}{{\bar{\varepsilon }}_{s}^{p}(\theta_{s},{\dot{\bar{\varepsilon }}}^{p})}=1$$

where $\omega_{s}$ is the state variable increasing with plastic deformation, and the plastic deformation is proportional to the equivalent plastic strain increment.

##### Interface

The interface between the fiber and the matrix is modeled with surface-based cohesive behavior (SBCB) interaction, the behavior of which is defined in terms of the traction–separation law. SBCB interaction is a less complex surface-based method to simulate the cohesive adhesion between fiber and matrix. The interface thickness is not considered. Although the accuracy of results can be improved by using cohesive elements, SBCB interaction is adopted, instead of cohesive elements, due to simulation time, which is also demonstrated, using a model effectively, in Refs. [3,38]

The cohesive damage initiates as follows:

(21) $${\left\{\frac{\langle t_{n}\rangle }{t_{n}^{0}}\right\}}^{2}+{\left\{\frac{t_{s}}{t_{s}^{0}}\right\}}^{2}+{\left\{\frac{t_{t}}{t_{t}^{0}}\right\}}^{2}=1$$

where $t_{n}^{0}$ represents the normal strength, $t_{s}^{0}$ and $t_{t}^{0}$ are the shear strengths, and $t_{n}$, $t_{s}$, and $t_{t}$ are the nominal vectors of traction. Additionally, $\langle t_{n}\rangle$ means that the pure compressive stress applied in the normal direction will not lead to the failure of the cohesive element.

Mixed-mode progressive damage is based on fracture energies, with the Benzeggagh-Kenane (BK) fracture criterion as follows [39]:

(22) $$G_{I}^{C}+\left(G_{\mathrm{II}}^{C}-G_{I}^{C}\right){\left\{\frac{G_{S}}{G_{T}}\right\}}^{\eta }=G^{C}$$

where $G_{S}=G_{II}+G_{III}$ and $G_{T}=G_{I}+G_{II}+G_{III}$. η is the mixed-mode parameter.

#### 2.4.2. Microscale Model Setup

**Figure 4:**
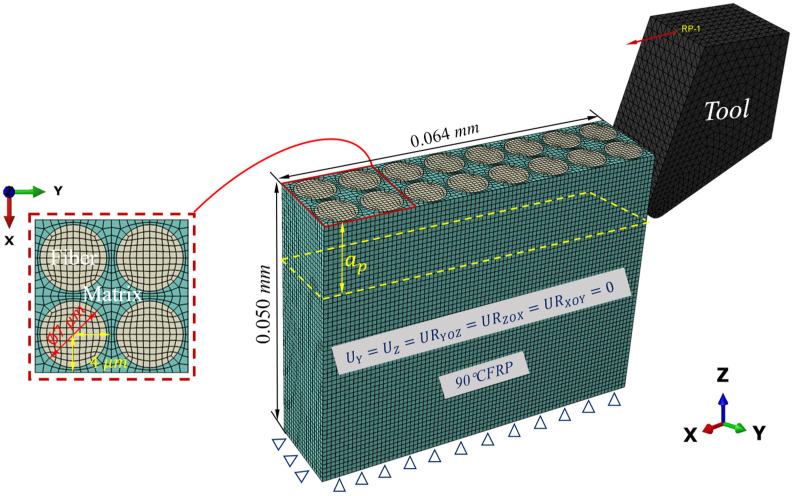
Microscale geometric model.

The geometric model in microscale is shown in Figure 4. The workpiece has a representative geometric size of 64 μm × 16 μm × 50 μm (length × width × height), and the cutting depth $a_{p}$ is 15 μm. A global mesh size of 0.8 μm  is adopted. The RVE and the tool are also meshed by C3D8RT. Additionally, the medial axis algorithm is adopted in the meshing method, ensuring consistent nodes across different components and enhancing computational efficiency. The pretreat temperature of both the workpiece and the tool are set based on the results of the macroscale model and experimental data. Figure 4 displays the representative volume element (RVE) with a fiber orientation of 90°, which can be adjusted to create various RVEs with different fiber orientations. The friction coefficient for fiber orientation at angles of 0°, 45°, 90°, and 135° are assigned values of 0.3, 0.6, 0.8, and 0.6, respectively. Similar to the macroscale model, the tool is regarded as a rigid body with a reference point RP to control its displacement. The cutting speeds are the same as in the experiments. The material parameters used in the microscale model are listed in Table 2.

## 3. Experimental Set Up

**Figure 5:**
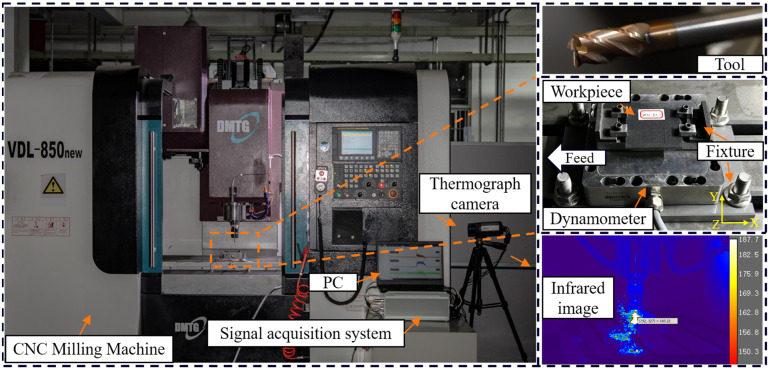
Experiment setup.

To ensure the accuracy of predictions made by the FE model, it is crucial to compare them with experimental results. This comparison helps to extend the numerical model to a wide variety of machining parameters and composite structures. The experimental setup is shown in Figure 5. The MD-CFRP laminates utilized in the experiment were produced by Shaanxi Tianqi New Composite Material Technology Co., Ltd., Xi’an, China, using the high-temperature mold pressing technique. Table 1 lists the material properties of T700/H69 ($T_{g}$ ≈ 180 °C) provided by the manufacturer. The MD-CFRP laminates are cut into 50 mm × 50 mm × 2 mm rectangular blocks, of which the lamination directions are ${\left[0^{{}^{\circ}},90^{{}^{\circ}},45^{{}^{\circ}},{-45}^{{}^{\circ}}\right]}_{2s}$. The fiber volume fraction is about 60%. To study the effects of cutting speeds and to guarantee the stability of heat transfer, a single factor milling experiment, including five different spindle speeds (1000, 2000, 4000, 6000, 8000 rpm) and a constant feed rate (1 m/min), was conducted with no coolant or lubricant. The milling experiments were conducted on a 3-axis CNC machine tool. The depth of the cut was 2 mm in the radius direction of the tool, and the thickness of the CFRP lamina in the axis direction. The experiments were repeated three times, and the results showed averaged values.

In the milling process, the workpieces were fixed by fixture upon the 3-axis force sensor. The sensor was fixed by a bottom plate attached to the machine tool worktable. The YG8 tools had a 15° rake angle and a 25° relief angle. Table 3 shows the properties of the YG8 tools. A ME (ME-K3D120) sensor, equipped with a charge amplifier (GSV-1A4), was utilized to measure the cutting forces and thrust forces. Real-time experimental data were collected using vDHDAS 6.20.9.9Zd collection software with a sampling frequency of 2 kHz. The cutting temperatures were measured using a high-speed infrared thermograph camera (Type FLIR A655) [41]. The typical infrared image recorded is also shown in Figure 5. After experiments, the scanning electron microscope (Type ZEISS Sigma 300) was used to observe the microscopic morphologies of the machined surfaces.

## 4. Result and Discussion

### 4.1. Cutting Force and Temperature Validation

**Figure 6:**
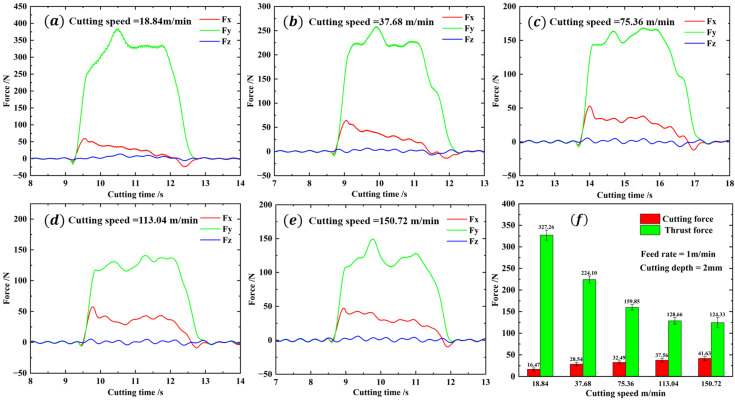
The force curves and average values of different cutting speeds. (**a**): The force curves at cutting speed of 18.84 m/min. (**b**): The force curves at cutting speed of 37.68 m/min. (**c**): The force curves at cutting speed of 75.36 m/min. (**d**): The force curves at cutting speed of 113.04 m/min. (**e**): The force curves at cutting speed of 150.72 m/min. (**f**): The average force values of different cutting speeds.

Figure 6a–e displays the force curves and average values of different cutting speeds. The graphs show that the cutting force and thrust force experience a rapid increase during the initial stage. Subsequently, the forces decrease slightly and fluctuate around a constant value. When the cutting process is completed, the cutting force and thrust force decrease back to their initial range. In the entire cutting time, the force in the Z direction fluctuates around 0. As shown in Figure 6f, the cutting force increases with the cutting speed, while the thrust force decreases with the increase in cutting speed. However, the rate of decrease in thrust force is considerably greater than the rate of increase in cutting force, and the rate of decrease in thrust force slows with the cutting speed increasing. When the cutting speed reaches 150.72 m/min, the cutting force obtained is the highest at 41.63 N, while the thrust force obtained is the lowest at 124.33 N. When the cutting speed is set to 18.84 m/min, the cutting force reaches a minimum value of 16.47 N, while the thrust force reaches a maximum value of 327.26 N.

**Figure 7:**
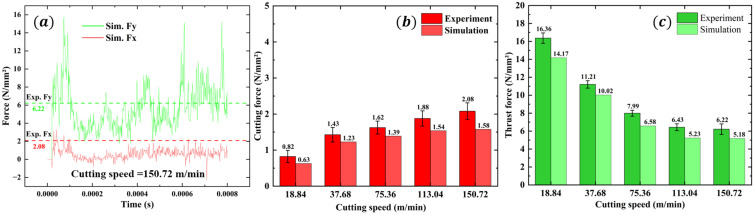
Experiment and simulation results of the forces at different cutting speeds. (**a**): The simulation force curves at cutting speed of 150.72 m/min. (**b**): The average cutting forces (Fx) of experiments and simulations at different cutting speeds. (**c**): The average thrust forces (Fy) of experiments and simulations at different cutting speeds.

Due to the computational efficiency, the geometry size of CFRP in the finite element model is different from that in the experiments. Therefore, the cutting force cannot be directly compared. In this paper, the cutting force is converted to per unit width and thickness. As depicted in Figure 7, the force results derived from the macroscale simulation closely correlate with experimental findings. The maximum error of cutting force and thrust force are 24% and 18%. Additionally, it is important to highlight that the simulation results are generally smaller than the experimental results. One reason is that, in the simulation, once the element reaches its failure strength, it is automatically deleted and no longer bears any force from any direction. Nevertheless, there are still some chips that survive in the fibers and matrix that are capable of withstanding force during the real cutting process. The other reason may be that tool wear is not considered in the simulation process. In the actual experiment, because the carbon fiber is hard in the cutting process, the friction and scratches with the tool cause tool wear to be very fast, resulting in a sharp increase in the cutting force, so the simulation values are smaller than the experimental values.

**Figure 8:**
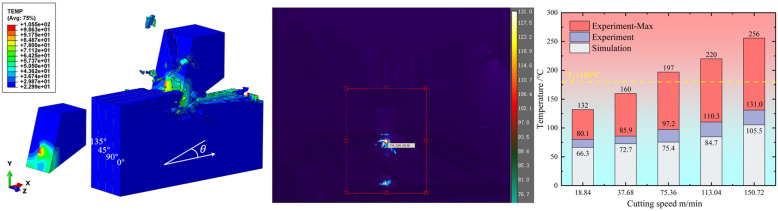
Experiment and simulation results of the cutting temperatures at different cutting speeds.

Figure 8 shows the cutting temperature results of the macroscale simulation and experiment; the simulation temperature results are in the same change trend as the experiment. As the cutting speed increases, both the simulation temperature and experimental temperature increase. While the cutting speed reaches 75.36 m/min, the maximum cutting temperature exceeds the glass transition point of 180 °C. When the cutting speed increases to 150 m/min, the cutting temperature reaches the maximum value of 256 °C. The maximum error of the cutting temperature is 23%. It is noted that the simulation temperature is slightly smaller than the experimental temperature, which may be due to the experiment process being inevitably affected by the environment, e.g., the air temperature, but the simulation model only considers the ideal situation. In addition, it can be seen from the temperature simulation result that the temperature distribution on the tool is uneven, which is caused by the different heat dissipation of the CFRP cutting process in different fiber directions. The temperature distribution area of the tool contact area, corresponding to 135° CFRP, is significantly less than that of the CFRP in other fiber directions, indicating that this heat dissipation is not sufficient and that the optimal heat dissipation area is the smallest, which is the same as the experimental conclusion obtained by Wang [4].

### 4.2. Surface Morphology

**Figure 9:**
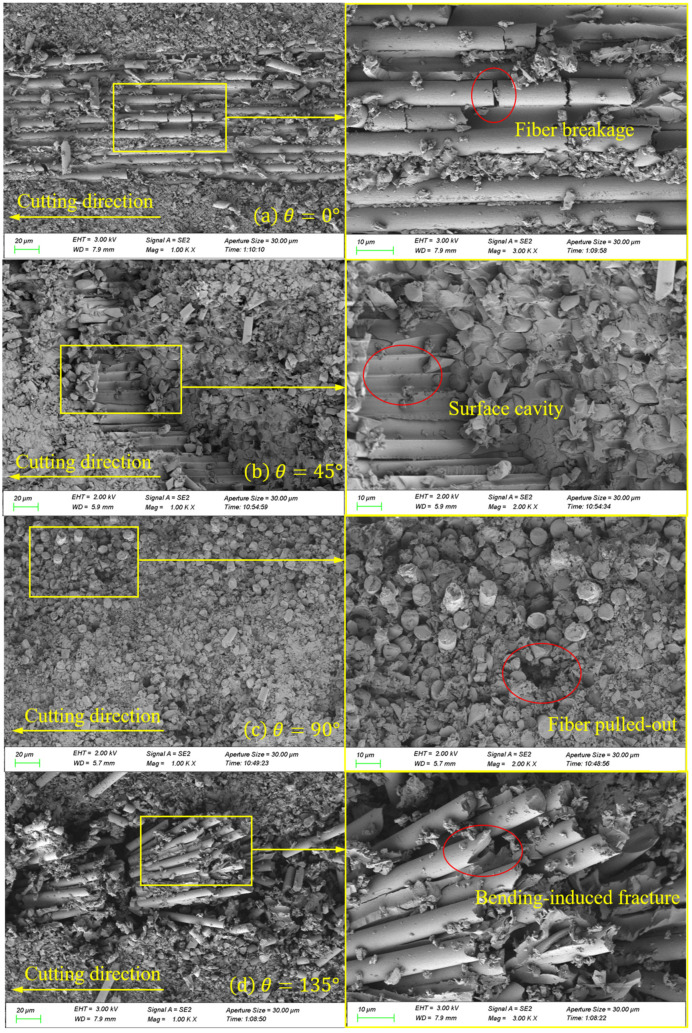
SEM images of four typical fiber orientations on a machined surface.

**Figure 10:**
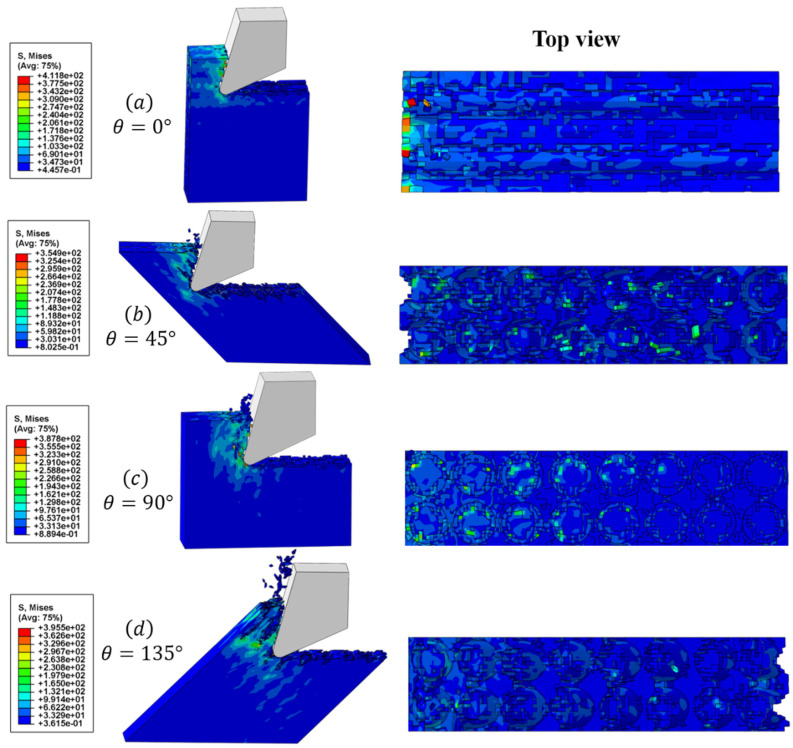
Cutting process of four typical fiber orientations and the resulting machined surface.

The SEM image results of the cross-section, after processing at a cutting speed of 150.72 m/min, are presented in Figure 9. In Figure 9a, the surface morphology of the 0° CFRP that was processed is displayed. Under the applied cutting load, the fiber is cut parallel to the cutting speed, resulting in a brittle fracture. This leads to the formation of a distinctive pull-off fracture, caused by a brittle removal. The fibers are uniformly and neatly arranged. A small portion of the fibers are directly broken by the tool, resulting in the removal of chips. However, the majority of the fibers are sheared and separated from the matrix, exposed to the processed surface. The image also shows fiber breakage, which could be attributed to the tool’s extrusion when cutting. Figure 9b shows the surface morphology of the 45° CFRP after processing. Under the effect of the cutting load, the fibers break when their strengths are exceeded. Additionally, the tooltip cuts some fibers directly in the radial direction, leaving cavity defects on the processed surface. This phenomenon was observed in Wang’s research [42]. It can be attributed to the material removal mechanism of the fiber bending, fracturing, and debonding from the matrix, which causes the fibers to be crushed and pulled out during the cutting process, resulting in surface cavity defects. Moreover, due to the inclination angle of the fiber, some fibers and resin debris remain in the long pits, as a result of the tool’s action and the obstruction caused by the fiber fracture. This leads to the filling of a portion of the pit depth, thereby contributing to the formation of a better processing surface. In Figure 9c, the surface morphology of the 90° CFRP after processing is depicted. The processed surface appears to be relatively flat and smooth, with a few fibers being pulled out. This can be attributed to the high cutting speed and low shear strength of the fibers, which allows for easier cutting along the radial direction and results in a neater fracture. It is observed that the fiber–matrix debonding phenomenon is not apparent, because the cutting mechanism is dominated by the shear effect and tool extrusion. In addition, the figure shows that a majority of the resin adheres plastically to the machined surface. This is because CFRP itself has poor thermal conductivity, preventing the heat generated during cutting from diffusing and cooling quickly. As a result, the broken resin is crushed under the combination of cutting heat and force, causing deformation and adherence to the processed surface. Figure 9d displays the surface morphology of the 135° CFRP after processing. Under the cutting load, the machined surface exhibits obvious unevenness and numerous fiber fractures. During the cutting process, the rake face of the tool initially comes into contact with the fiber, causing bending stress on the upper part of the fiber as the tool advances. When this bending stress surpasses the shear strength of the fiber, the upper part of the fiber breaks, resulting in an actual cutting thickness that is less than the cutting depth. Furthermore, the inclination of the cutting angle and the anisotropic mechanical properties of the fiber itself contribute to an uneven fracture of the fibers. Due to the small heat dissipation area [4], a large amount of cutting heat is difficult to dissipate, causing the resin matrix to melt and adhere to the surface of the fiber, ultimately leading to poor surface quality. Figure 10 shows the machined surface simulated by microscale model. From the comparison of the figures, it can be seen that the machined surface morphology is generally consistent with the experiment results.

### 4.3. Cutting Mechanism under Thermal Effect

**Figure 11:**
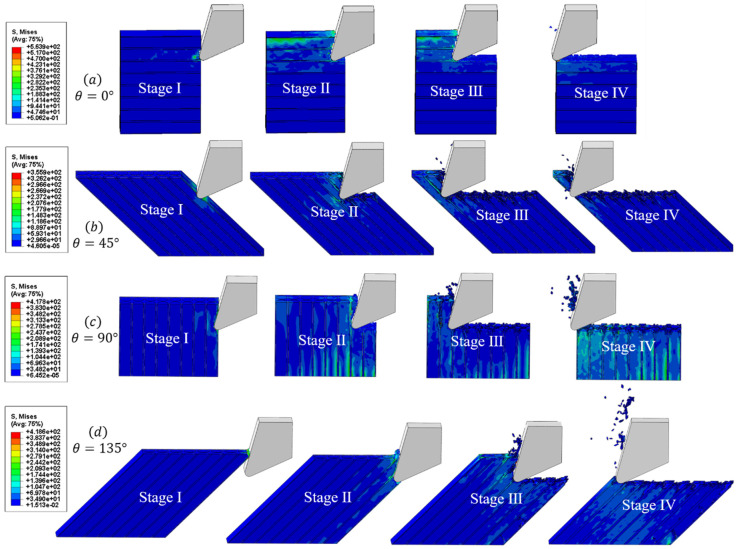
The stress change in typical fiber orientations during the CFRP cutting process at 150.72 m/min and under 256 °C.

**Figure 12:**
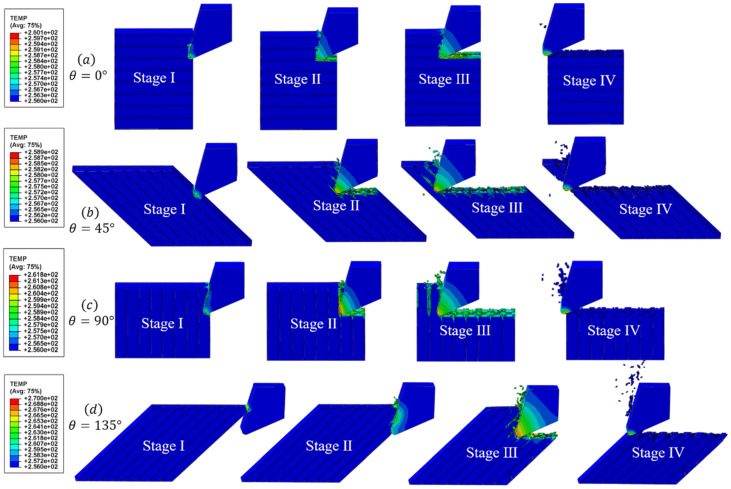
The temperature change in typical fiber orientations during the CFRP cutting process at 150.72 m/min and under 256 °C.

As shown in Figure 11 and Figure 12, the microscopic model results reveal four typical fiber orientations resulting from a CFRP cutting process at 150.72 m/min and under 256 °C, which can be divided into four stages.

Stage I. In the initial stage, as the tooltip makes contact with the workpiece, stress is concentrated in the cutting contact area and moves forward in the direction of the tool’s cutting speed. The heat generated is primarily due to the material’s deformation and fracturing, resulting in less heat generation and a slower temperature rise. Additionally, the high thermal conductivity of the tool material causes the temperature to be mainly concentrated on the tool side. When the cutting angle is less than 135°, the temperature is mainly concentrated at the tooltip. However, when the fiber orientation is set to 135°, the temperature is concentrated on the rake face of the tool as it makes contact with the workpiece first.

Stage II. In the second stage, the tool continues to move forward. The rake face of the tool comes into contact with the workpiece, resulting in stress being transmitted downwards and forwards along the axial direction of the fiber. The main heat source is generated by the friction between the tool rake face and the workpiece, and the temperature gradually conducts from the tooltip to the outer surface, as explained in Section 2.2.

Stage III. In the third stage, as the tool progresses into the stable cutting stage, a significant amount of heat is generated due to the friction between the rake face and the chips, as well as the friction between the flank face and the machined surface. The temperature in the cutting area rises rapidly and spreads along the fiber direction, concentrating at the fiber–matrix interface. At this time, the temperature exceeds the glass transition temperature of the resin matrix, causing it to melt and be unable to support and fix the fibers. As a result, the fibers are cut and extruded to form some chips that flow out with the rake face. Under the extrusion of the flank face, another portion of the chips are coated on the machined surface, forming stress and temperature residues.

Stage IV. In the final stage, a large number of chips are removed with the rake face to form a machined surface. A small amount of heat residue remains at the tooltip due to friction between the flank face and the machined surface. The temperature of the cutting area decreases with the ambient cooling temperature, and there is a small amount of the spring-back phenomenon in the matrix and the fiber after processing. Defects, such as burrs on the machined surface, fiber pull-out, and fiber breakage under the machined surface, can be seen in the figure.

Moreover, it should be emphasized that, for 0° fiber direction cutting, only a small number of chips are generated, due to the high cutting speed and the dominance of fiber bending and tool extrusion in the cutting process. Additionally, when the cutting speed and predefined temperature are the same, the 45° fiber orientation CFRP shows the lowest cutting temperature, whereas the 135° fiber orientation CFRP exhibits the highest cutting temperature. This result is consistent with Wang’s conclusion [4], which can be attributed to the difference in the size of the heat dissipation zone.

## 5. Conclusions

This paper presents coupled thermal–mechanical models, which are both macroscale and microscale, to investigate the effect of heat generation and material removal mechanisms in the machining of CFRP. Experiments have confirmed the reliability and predictability of the macroscale model. Based on the above outcomes, researchers and workers can better choose the cutting parameters and design the stacking sequence of CFRP, to achieve low-damage manufacturing. The following conclusions can be drawn from the study:

1. The cutting force increases with a higher cutting speed, while the thrust force decreases with a cutting speed increase. In the cutting process, the thrust force is greater than the cutting force, and the rate of decrease in thrust force is greater than the rate of increase in cutting force. Considering the requirements of actual processing and production, it is recommended to use higher cutting speeds.
2. The cutting temperature increases as the cutting speed increases. When the cutting speed reaches 70.36 m/min, the maximum cutting temperature exceeds the glass transition temperature of the resin. This causes the resin to melt and become unable to support the fiber, resulting in poor surface quality.
3. The process of cutting CFRP can be divided into four stages. In the initial stage, stress concentration occurs due to contact between the tool tip and the workpiece. During this stage, heat is primarily generated through the deformation and fracturing of the workpiece material and is concentrated in a small area around the cutting edge. As time progresses, heat generation converts to be due to friction between the tool and the workpiece, resulting in the temperature of the tool increasing rapidly and diffusing to a wider area due to its higher thermal conductivity.
4. The temperature significantly affects the mechanism and process of removing CFRP materials. Additionally, the cutting process of CFRP varies depending on the orientation of the fibers. Heat diffuses along the fiber direction during the cutting process, leading to debonding between the fiber and the matrix, which further causes the occurrence of fiber pull-out, burrs, and the initiation of cracks.
5. The macroscale model proposed in this paper has a good predictability for cutting forces and cutting temperatures. It can guide actual processing and production. The microscale model explains the cutting mechanism of CFRP under thermal–mechanical coupling, which can help other scholars understand the process more vividly and easily.
6. In this work, tool wear was not considered, and the fibers were regularly arranged in the matrix. In actual machining, tool wear is inevitable. In addition, it can be seen that the actual cross-section of CFRP shows random distribution of carbon fibers in the resin, from SEM images. These concepts will be considered in future models to further improve the accuracy of the model and more accurately simulate the real cutting process.

## Figures and Tables

**Table 1:** Material properties of the macroscopic model.

Parameters	Values
Density $\rho \;(kg/m^{3})$	1570
Poisson’s ratio v	0.3
Glass transition temperature $T_{g}\;\left({}^{\circ}C\right)$	180
Longitudinal Young’s modulus $E_{1}\;(GPa)$	153
Transverse Young’s modulus $E_{2}\;(GPa)$	10.3
Shear Young’s modulus $G_{12}\;(GPa)$	6
Shear Young’s modulus $G_{23}\;(GPa)$	3.7
Longitudinal tensile strength $\;X^{T}\;(MPa)$	2537
Longitudinal compression strength $\;X^{C}\;(MPa)$	1580
Transverse tensile strength $\;Y^{T}\;(MPa)$	51
Transverse compressive strength $\;Y^{C}\;(MPa)$	130
Shear strength $S^{L}\;(MPa)$	90
Shear strength $S^{T}\;(MPa)$	75
Specific heat C (J/(kg·K)) [25]	1200
Longitudinal thermal conductivity $\lambda_{1}\;(W/(m\cdot K)$ [25]	7
Transverse thermal conductivity $\;\lambda_{2}\;(W/(m\cdot K))$ [25]	0.8
Longitudinal thermal expansion coefficient $\alpha_{1}\;(10^{-6}/K)$ [37]	26
Transverse thermal expansion coefficient $\;\alpha_{2}\;(10^{-6}/K$) [37]	1

**Table 2:** Material properties of microscopic model [17,20,40].

Material	Property	Values
Carbon fiber	Density	$\rho_{f}$ = 1.76 $\times 10^{-9}$ (ton/${mm}^{3}$)
	Elastic constants	$E_{11}$ = 231 GPa,$\;E_{22}\;$= ${\;E}_{33}\;$= 15 GPa, $\nu_{12}\;$=$\;\nu_{13}\;$= 0.2, $\nu_{23}\;$= 0.25, $G_{12}\;$= ${\;G}_{13\;}$= 15 GPa, $G_{23}\;$= 7 GPa
	Tensile strength	$X_{t\;}$= 3590 MPa, $Y_{t}\;$= 350 MPa
	Compressive strength	$X_{c}\;$= 1800 MPa, $Y_{c}\;$= 2730 MPa
	Shear strength	S = 380 MPa
	Longitudinal thermal conductivity	$\lambda_{l}\;$= 9.37 W/m ·°C
	Transverse thermal conductivity	$\lambda_{t}\;$= 4 W/m ·°C
	Longitudinal thermal expansion	$\alpha_{l\;}\;$= −0.7 $\times 10^{-6}$/°C
	Transverse thermal expansion	$\alpha_{t\;}$= 12 $\times 10^{-6}$/°C
	Specific heat	$C_{f}\;$= 7.94 $\times 10^{8}\;m$J/(ton°C)
Matrix	Density	$\rho_{m}\;$= 1.2 $\times 10^{-9}$ (ton/${mm}^{3}$)
	Elastic constants	$E_{m}\;$= 3.35 GPa, $\nu_{m}\;$= 0.35
	Yield strength	$\sigma_{y0}^{m}\;$= 120 MPa, ${\bar{\varepsilon }}_{f}^{pl}$ = 0.05
	Johnson–Cook parameter	A = 120 MPa, B = 654.18 MPa, n = 0.772, c = 0.124, m = 0.304
	Thermal conductivity	$\lambda_{m\;}$= 0.36 W/m ·°C
	Thermal expansion	$\alpha_{m\;}$= 5.8 $\times 10^{-5}$/°C
	Specific heat	$C_{m}\;$= 1.31 $\times 10^{9}\;m$J/(ton°C)
	Fracture energy	$G_{m}^{c}\;$= 0.1 N/mm
Interface	Normal strength	$t_{n}\;$= 50 MPa
	Shear strength	$t_{s}\;$= 75 MPa
	Elastic stiffness	K = 100,000 N/${mm}^{3}$
	Fracture energy	$G_{I}^{C}\;$= 0.002 N/mm $G_{II}^{C}\;$= ${\;G}_{III}^{C}\;$= 0.006 N/mm
	Mixed-mode parameter	η=1.45

**Table 3:** Properties of YG8 tool.

Parameters	Values
Density	1.45 × $10^{-8}$ (ton/${mm}^{3}$)
Specific heat	3.94 × $10^{8}\;m$J/(ton°C)
Thermal conductivity	42 W/m ·°C
Thermal expansion	4.5 × $10^{-6}$/°C
Young’s modulus	600 GPa
Poisson’s ratio	0.24
Rake angle	15°
Relief angle	25°
Helix angle	45°
Tool diameter	6 mm
Radius of cutting edge	2.5 $\times 10^{-3}$ mm

## Data Availability

Not applicable.
